# Aponeurosis linguae—Myocutaneous or myotendinous junctions of skeletal muscle fibres in the human tongue?

**DOI:** 10.1111/joa.13637

**Published:** 2022-02-08

**Authors:** Christian Albrecht May

**Affiliations:** ^1^ Department of Anatomy Medical Faculty Carl Gustav Carus, TU Dresden Dresden Germany

**Keywords:** insertion, skin, striated muscle, tongue

## Abstract

The morphology of the more superficial tissue of the human tongue was investigated and discussed with the clinical appearance of fissures. Three regions could be distinguished according to the presence and shape of the aponeurosis linguae: the central region showed a thick aponeurotic plate with myotendinous muscle fibre insertions. The lateral region showed still an aponeurosis linguae but of reduced thickness and without muscle insertions. The edge‐wise and lower region showed no aponeurosis linguae but a fatty subcutis and myocutaneous muscle fibre insertions lacking specific molecules of myotendinous junctions. This system of partially developed exoskeleton seems to underlie but not to be involved in tongue fissures, which are more superficial within the epidermis and dermis.

## Graphical abstract

The superficial tissue of the human tongue shows three distinct regions: 1. As seen in the figure, the central region shows a thick aponeurosis linguae and myo‐tendinous insertions of the muscle fibers. 2. The lateral region showed a reduced aponeurosis and no muscle insertions. 3. The edge‐wise and lower region showed no aponeurosis but myo‐cutaneous junctions.

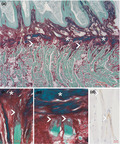

## 1 INTRODUCTION

The tongue is a diagnostic tool for numerous conditions and diseases. One aspect is the landform of the surface area distinguishing plain from notched appearances, the latter showing different orientations and locations of fissures (described in more detail in the discussion). It is still a matter of debate so to how the fissures develop and vanish (since they are often temporary in nature) and which morphological aspects are related to this appearance. To address this question, I focused on the detailed morphology of the tongue tissue underneath the mucosa.

As an exception to the majority of skeletal muscle fibres in the human, the tongue is characterized by a fibrous exoskeleton (aponeurosis linguae) located immediately under the covering mucosa and merging with the subcutaneous connective tissue (dermis). The mucosa can be described as a specialized stratified squamous epithelium forming at places specific papillae linguales. The dermis contains loosely arranged collagen fibres with numerous blood vessels and small nerve fibre bundles. Depending on the region, the papillary dermis forms either lank finger‐like protrusions of different lengths (dorsum linguae) or clumsy and short protrusions (lateral regions) or even no obvious protrusions (ventral surface). The aponeurosis is thickest near the sulcus terminalis and becomes thinner towards the tip and the sides of the tongue. It fades away at the margins of the tongue and dorsal of the sulcus terminalis. The collagen fibres form a dense meshwork running parallel to the surface; this meshwork changes its appearance such as a folding grille when the tongue stretches (Dontenwill, 1949). The internal muscle bundles of the tongue insert from deep towards this fibrous layer which is closely connected to the epithelium covering the tongue. Only recently, I described a specific morphological appearance of the myocutaneous junctions present in some mimic muscles (May & Bramke, 2021). The finger‐like protrusions at the ending of single muscle fibres were less strictly parallel and lacked some specific structures (e.g. collagen XXII) usually present at myotendinous junctions. The aim of the present report is to describe the junctional side of the muscle fibres in the human tongue and its interaction with the three‐dimensional architecture of the aponeurosis linguae.

## 2 MATERIALS AND METHODS

### 2.1 Tissue preparation

Specimens of the tongue were collected from five human cadavers. They were part of the donor programme of the Department of Anatomy in Dresden (Germany) and had given in their lifetime written consent that their body might be used for the purpose of science and education after death. There were two male and three female cadavers, the age range was between 76 and 92 years, lacking neuro‐ or myopathies in their medical history as far as documented.

The whole tongues were resected 1–2 days post‐mortem and further dissected in different parts: the ventral 1.5 cm were defined as apex linguae. A posterior 1 cm wide strip was cut from one side to the other with its dorsal margin at the sulcus terminals; this strip was further divided into equal‐sized medial, intermedial and lateral parts. A second strip was cut in the middle, between the posterior strip and the tip, and also divided into three parts. All tissue samples were fixed in 4% neutral buffered formalin for 24 h. The samples were washed several times in phosphate‐buffered saline (PBS, pH 7.4, 0.01 M) and then processed for embedding in paraffin wax.

### 2.2 Histology and immunohistochemistry

Serial sections (5 μm thick) of each specimen were performed in different planes and selected sections were stained with haematoxylin and eosin (H&E) and Sirius red solution.

For immunohistochemistry, consecutive sections of H&E stained sections containing ends of muscle fibres were dewaxed, rehydrated and irradiated with microwaves in 0.01 M sodium citrate buffer (pH 6.0) for 2 × 5 min at 800 W to unmask the antigens. After washing in PBS, the sections were treated with 0.3% hydrogen peroxide for 10 min and blocked in normal mouse serum for 15 min at 37°C followed by washing in PBS. The primary antibody (rabbit against Collagen XXIIA1 [aa 181–273; Creative Diagnostics; dilution 1:100], mouse against desmin [Quartett, dilution 1:10], mouse against vinculin [Serotec, dilution 1:100], mouse against beta1 integrin [Novo Castra, dilution 1:100]) was applied overnight at 4°C. After washing in PBS, an appropriate biotinylated secondary antibody was added and incubated for 15 min at 37°C, followed by washing and incubation with a VECTASTAIN® Elite ABC mouse kit (PK 6101; PK 6102 Vector Laboratories Inc.). Visualization of peroxidase activity was realised by adding 3,3‐diaminobenzidine for 8 min.

The sections were examined on Zeiss Jenamed2 microscope (Carl Zeiss AG) and images were recorded using a Digital Sight DS‐Fi1 camera (Nikon AG).

## 3 RESULTS

Three distinct regions could be differentiated and were investigated separately: the central upper region of the tongue showing a well‐developed aponeurosis linguae, the lateral and rim region with a thin aponeurotic layer and the edge‐wise and lower regions without aponeurotic tissue. These regions showed distinct morphological features and measurements (Table 1).

**TABLE 1:** Thickness of different layers in different regions of the tongue

	Central region (Figure 1)	Lateral region (Figure 2)	Inferior region (Figure 3)
Epithelial thickness (min–max in μm)	80–100	80–160	120–200
Connective tissue papilla (maximal height in μm)	1600	2000	40
Reticular dermis thickness (min–max in μm)	300–350	160–260	140–190
Thickness of aponeurosis linguae (min–max in μm)	280–320	100–150	Not present

### 3.1 Central upper region (Figure 1)

**FIGURE 1:**
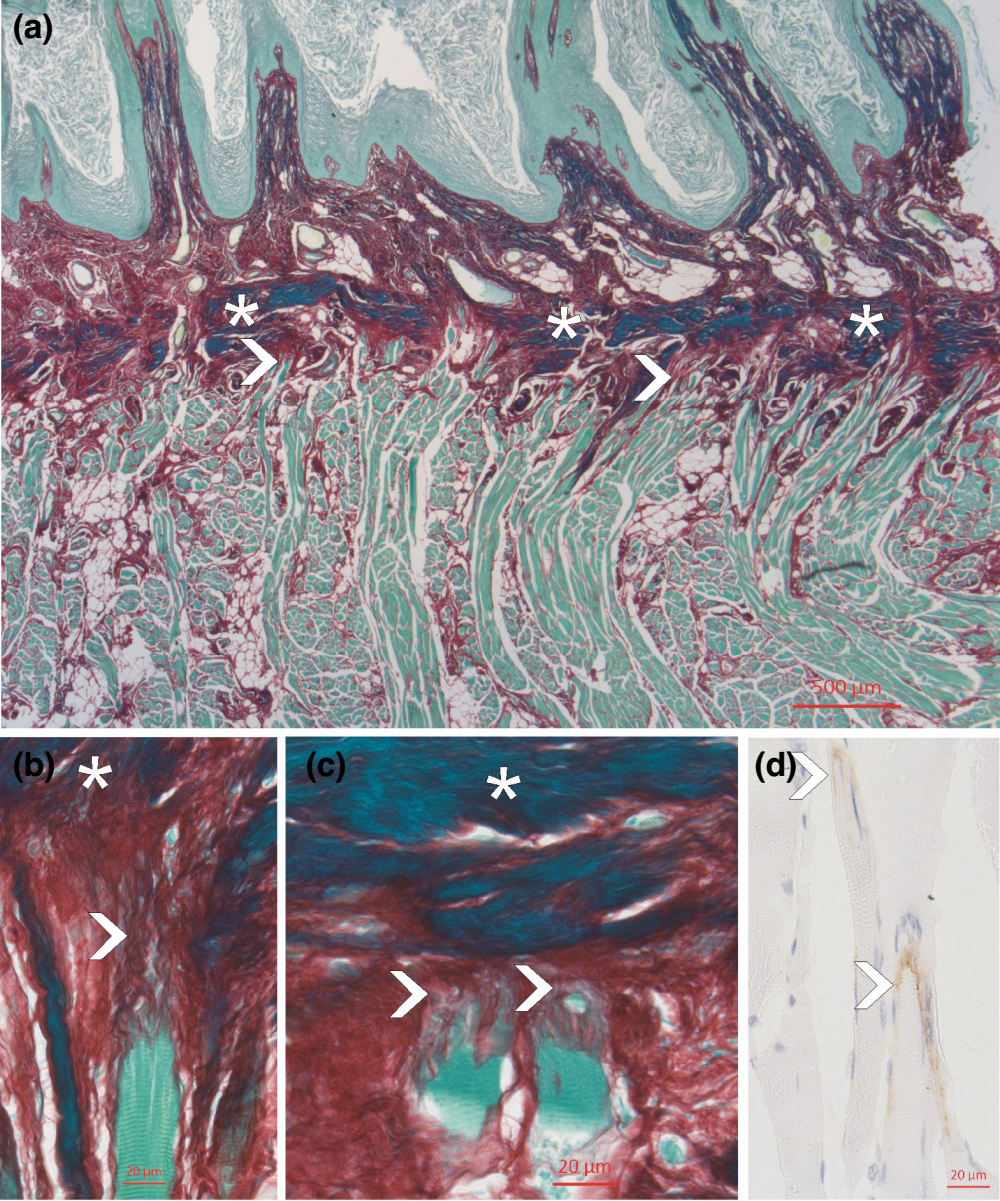
Sagittal section through the central upper region of the tongue, stained with Sirius red (a–c). Note the aponeurosis linguae stained dark green (asterisks) and the insertion of the striated muscle fibres (arrowheads) either with small tendons (b) or directly towards the aponeurosis (c). The myotendinous junctions showed staining for collagen type XXII (brown colour in d)

In this region, the aponeurosis linguae was fully developed and could be clearly distinguished as a dense collagen plate in contrast to the more loosely arranged tissue of the dermis which was highly vascularized. Due to the dense collagen bundles, the aponeurosis stained dark green with the Sirius red staining. The 280–320‐μm‐thick aponeurosis showed regular ‘gaps’ for the vessels and nerves to enter the dermis. Only occasionally striated muscle fibres joined these gaps and reached the outer border of the aponeurosis. Most of the striated muscle fibres ended at the inner border of the aponeurosis with a typical finger‐like appearance. At some places, the joining collagen fibres formed loose tendon‐like formations (Figure 1b), at other places, these tendon‐like structures were not visible and the collagen bundles joined immediately the aponeurosis (Figure 1c). The endings of the muscle fibres stained positive for collagen XXII.

### 3.2 Lateral and rim region (Figure 2)

**FIGURE 2:**
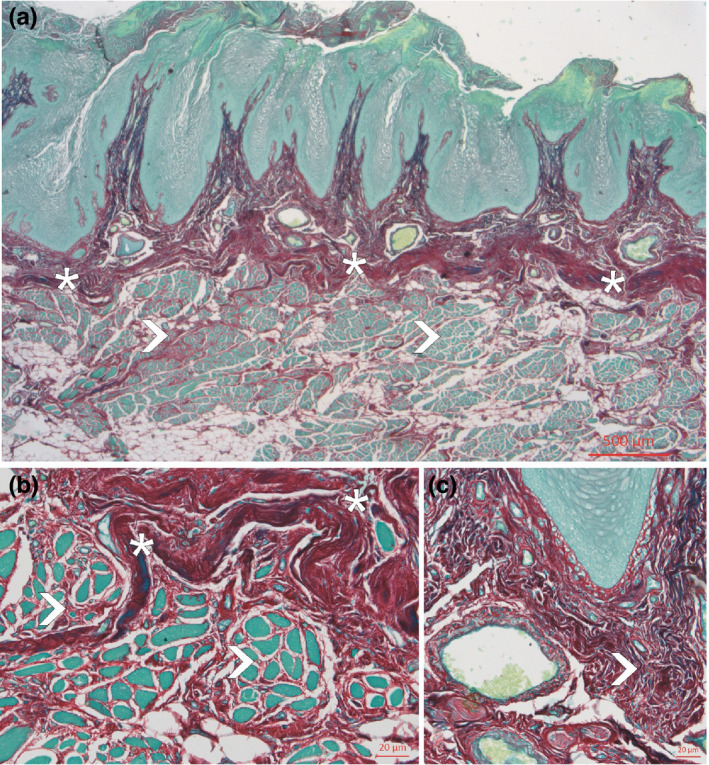
Frontal section through the lateral and rim region of the tongue, stained with Sirius red. The reduced aponeurosis linguae (asterisks) stains similar to the surrounding connective tissue dark red. The skeletal muscle bundles of the tongue (arrowheads in a and b) show no insertion towards the aponeurosis. (c) The dense connective tissue of the dermis (arrowhead) filled with vessels and nerves

In this region, the aponeurosis linguae was less compact and showed the normal red staining for collagen tissue usually present with the Sirius red stain. The aponeurosis linguae was also thinner than in the central region, but the connective tissue papillae were somewhat elongated (Table 1). The dermis was highly vascularized and showed numerous larger veins. Large bundles of striated muscles touched the inner border of the aponeurosis but in this region, there were no muscle fibre endings.

### 3.3 Edge‐wise and lower region (Figure 3)

**FIGURE 3:**
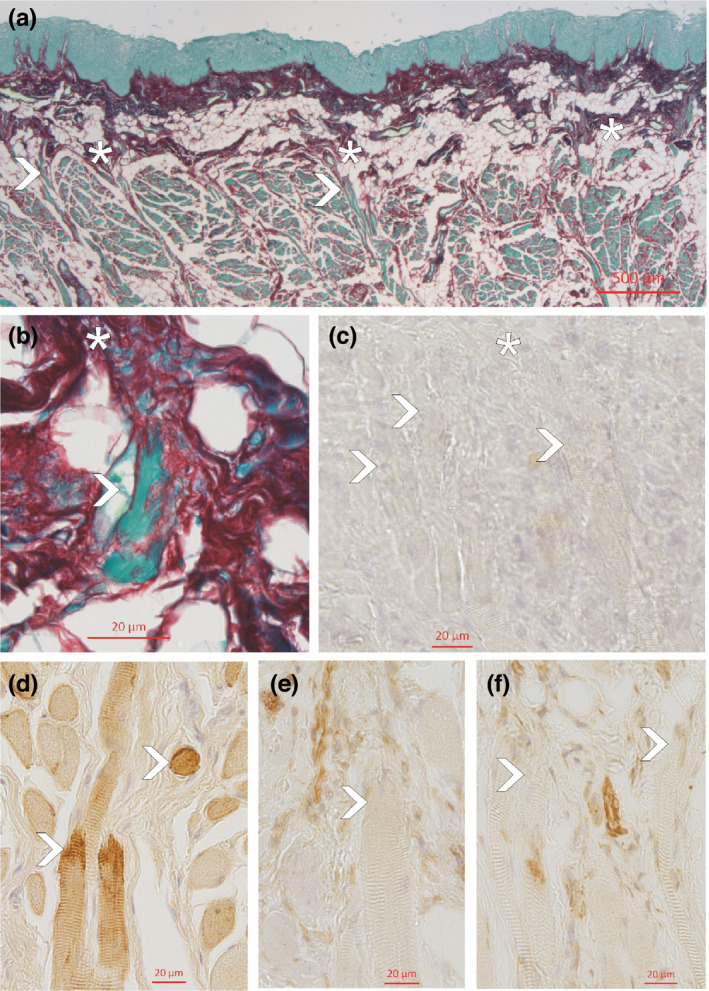
Frontal section through the edge‐wise and lower region of the tongue, stained with Sirius red (a and b). Note the lack of a continuous aponeurosis linguae (asterisks) and the high amount of fatty tissue (white areas). The insertion of the striated muscle fibres (arrowheads) shows no tendon formation but rather a diffuse communication with the dermal layer (b). The myocutaneous junctions did not stain for collagen type XXII (c). They showed some accumulation of desmin (d) but no accumulation of either beta1 integrin (e) or vinculin (f)

In this region, there were no aponeurosis linguae. The thin dermis (Table 1) merged with fatty tissue which was partly filled with seromucous glands. At places, small bundles of striated muscle fibres entered the dermis in a rectangular position and ended mainly without forming tendon‐like collagen connections (Figure 3b). All endings in this region stained negative for collagen XXII (Figure 3c). There was some positive staining for desmin at these endings (Figure 3d), but beta1 integrin (Figure 3e) and vinculin (Figure 3f), both markers for myotendinous junctions, were also negative.

## 4 DISCUSSION

Hitherto, no morphological differentiation of different tongue regions is established. Although it is known that the aponeurosis linguae covers only part of the muscular body of the tongue (Dontenwill, 1949), ultrastructural studies of the muscle fibre insertion (performed with guinea pigs) did not differentiate between insertion sides (Demmel et al., 1979; Hanak & Böck, 1971). The present study demonstrated that there are two insertion regions of tongue striated muscle fibres that can be distinguished: one is in connection with the aponeurosis linguae and develops characteristic features seen in myotendinous junctions including the expression of collagen XXII. The other insertion zone of muscle fibres is outside the aponeurosis linguae where the muscle fibres enter the dermis and are comparable to the newly described myocutaneous junctions with a lack of collagen XXII expression. While the latter connection could influence the mucous surface area more directly and locally, the myotendinous connection at the aponeurosis linguae distributes their effect towards the whole exoskeletal plate. In addition, the dermis in regions with myocutaneous junctions shows a fatty subcutaneous tissue which allows more movement of the dermal layer, while at the aponeurosis linguae, the dermis is strongly fixed to the aponeurosis with bundles of collagen and thus firmly fixed to the base. The different types of muscle insertion might parallel tongue movement describing it towards a more cutaneous movement in the lateral and extrusive direction, and a more skeletal movement when lifting the tongue.

Could the tongue muscles and aponeurosis be involved in the clinical sign of fissure formation? Most clinical studies do not address the question of morphological ability but rather distinguish the fissures of the tongue due to their location and appearance. Many names were introduced: cerebriform tongue, lingua fissurata, lingua plicata, lingua scrotalis, geographic tongue, to name the most frequent terms. Two characteristic patterns can be stated when comparing the literature: a fissured tongue with larger notches in the region of the aponeurosis linguae, typically ventral‐dorsal oriented (saggital fissures; Sudarshan et al., 2015), and a geographic tongue with irregular areal notches more towards the lateral regions of the tongue, typically not as deep as the fissures. Different factors are associated with these two patterns (Dafar et al., 2016). The geographic tongue is characterized by the loss of filiform papillae and by habitual factors, for example chewing tobacco (Assimakopoulos et al., 2002; Dafar et al., 2016); the genesis of fissures is unknown, discussing genetic factors and systemic diseases (Dawson, 1974; Eidelman et al., 1976; Guggenheimer et al., 2000; Hamrah et al., 2021; Marcoval et al., 2012; Monshi et al., 2021). The papillae in fissured tongues are preserved and the tongue shows a higher accumulation of inflammatory cells (Kullaa‐Mikkonen & Sorvari, 1986). The morphological changes seem to be limited to the dermis and point to an edematous stage (Järvinen et al., 2014; Kullaa et al., 1993). This could explain the disappearance of tongue fissures after successful treatment of systemic inflammations. The aponeurosis linguae and the inserting striated muscle fibres seem not to be involved in this condition. Clinical changes of the aponeurosis might be rather metaplastic towards cartilaginous tissue (Munro & Singh, 1990; Pereira et al., 2012; Takeda, 1987; Toida et al., 2003; van der Wal & van der Waal, 1987).

## Data Availability

Not sharing any other data than reported.
